# A Hierarchical Bayesian approach to small area estimation of health insurance coverage in Ethiopian administrative zones for better policies and programs

**DOI:** 10.1186/s13561-024-00498-3

**Published:** 2024-04-16

**Authors:** Yegnanew A. Shiferaw, Seyifemickael Amare Yilema, Yikeber Abebaw Moyehodie

**Affiliations:** 1 Department of Statistics, University of Johannesburg, PO Box 524, Johannesburg, 2006 South Africa; 2 Department of Statistics, College of Natural and Computational Science, Debre Tabor University, PO Box 272, Debre Tabor, Ethiopia

**Keywords:** Disaggregated level CBHI scheme, Hierarchical Bayes model, Small area estimation, Ethiopia

## Abstract

Sample surveys are extensively used to provide reliable direct estimates for large areas or domains with enough sample sizes at national and regional levels. However, zones are unplanned domains by the Demographic and Health Survey (DHS) program and need more sample sizes to produce direct survey estimates with adequate precision. Conducting surveys in small areas (like zones) is too expensive and time-consuming, making it unfeasible for developing countries like Ethiopia. Therefore, this study aims to use the Hierarchical Bayes (HB) Small Area Estimation (SAE) model to estimate the Community-Based Health Insurance (CBHI) coverage at the zone levels in Ethiopia. To achieve this, we combined the 2019 Ethiopia Mini-Demographic and Health Survey (EMDHS) data with the 2007 population census data. SAE has addressed the challenge of producing reliable parameter estimates for small or even zero sample sizes across Ethiopian zones by utilizing auxiliary information from the population census. The results show that model-based estimates generated by the SAE approach are more accurate than direct survey estimates of CBHI. A map of CBHI scheme coverage was also used to visualize the spatial variation in the distribution of CBHI scheme coverage. From the CBHI scheme coverage map, we noticed notable variations in CBHI scheme coverage across Ethiopian zones. Additionally, this research identified areas with high and low CBHI scheme coverage to improve decision-making and increase coverage in Ethiopia. One of the novelties of this paper is estimating the non-sampled zones; therefore, the policymakers will give equal attention similar to the sampled zones.

## Introduction

Health insurance is vital to the realization of universal healthcare and the prevention of disease. Community-based health insurance (CBHI) is the most effective means to provide universal health coverage (UHC) with enough financial protection against healthcare expenses, promote equitable access to high-quality healthcare, raise financial security, and improve social cohesion and solidarity [1–3]. Out-of-pocket medical expenses, however, lead to lower healthcare service utilization and higher catastrophic medical expenses. Certain countries have implemented CBHI programs to reduce out-of-pocket expenditures and enhance access to healthcare services, particularly for people living in rural areas or working in the unorganized sector [4, 5].

Around 100 million people are forced into extreme poverty yearly due to out-of-pocket health spending, and over 930 million individuals worldwide spend at least 10% of their household income on healthcare [6]. CBHI is becoming more popular in low- and middle-income countries to enhance healthcare utilization and shield households from financial catastrophes brought on by out-of-pocket medical charges [7]. A significant issue in Africa has been the catastrophic nature of healthcare financing arrangements for the poor, particularly the rural population [8]. Out-of-pocket charges can significantly hinder access to quality medical treatment in Sub-Saharan African nations[9]. Most sub-Saharan African nations, including Ethiopia, have adopted the CBHI program to increase the population's fair access to health services. However, enrollment rates and renewals for CBHI in sub-Saharan Africa are persistently low [10].

In 2019, it was estimated that 28% of the Ethiopian population nationwide had health insurance, primarily community-based health insurance [11, 12]. In other words, nearly 82 million Ethiopians, or about 72% of the country's total population, lacked health insurance enrollment. According to [13], a lack of health insurance is significantly associated with worse health outcomes and restricted access to medical care. People without insurance may experience health problems due to their lack of coverage. This could also affect the quality and organization of local healthcare systems [14].

The Sustainable Development Goal for Health (SDG3) aimed to ensure healthy lives and promote well-being for people of all ages [15]. SDG3 includes a sub-target (3.8) on achieving UHC, including financial risk protection and access to quality, essential health services. One of the SDGs for 2030 is UHC, which means everyone can access a full range of high-quality health services when and where they need them without financial hardship [16]. This goal still needs to be accomplished in six years. Although the UHC service coverage index (SDG indicator 3.8) has increased from 45 in 2000 to 67 in 2019, 30% of the world's population still lacks access to essential health services [17]. Ethiopia's health insurance coverage in 2019 was 28% [12], far below the SDG target of 80% by 2030. The inability to reach other SDG health-related targets results from this low coverage rate compared to other nations in the region. Additionally, 31% of all medical spending in 2016–17 was attributed to catastrophic out-of-pocket costs, which have remained high over time [18].

To achieve UHC in Ethiopia, it is essential to understand the impact of health insurance on healthcare utilization and financial protection. Studies have highlighted the potential factors related to maternal health-seeking behavior and the association between health insurance membership and antenatal care utilization in Ethiopia [19]. Additionally, the impact of CBHI on universal health coverage in Ethiopia has been evaluated, indicating positive impacts on health service issues by reducing catastrophic costs. However, the low universal health coverage for a limited population suggests a broader national scheme covering the entire population [20].

Aside from that, coverage varies according to administrative regions and other sociodemographic factors [12]. According to a recent report by [17], despite improvements in healthcare coverage nationally, intra-country disparities are obscured by aggregate data, making it challenging for UHC to address inequalities.

The studies above compiled national and regional health insurance coverage estimates through household surveys. These surveys are used to obtain accurate statistical information about a specific target population in broad regions, such as the nation, the state, the province, and so on [21, 22]. However, estimates obtained from local surveys are often unreliable due to inadequate sample sizes. In the case of Ethiopia, estimates for lower administrative levels, such as zones, are unavailable due to small sample sizes. To address this issue and produce more precise estimates, the small area estimation (SAE) model combines survey and census data.

Small areas refer to subpopulations or unplanned domains in large-scale sample surveys. In these cases, model-based methods, also known as the SAE approach, have consistently attracted attention for their ability to provide appropriate estimates for such a small area or domain. The 2030 Agenda for Sustainable Development Goals (SDGs) emphasizes the importance of decentralized statistics for micro-level planning, policy formation, and targeted social advancement. SAE is indispensable for reconciling the demand for reliable, representative, and disaggregated official statistics [21].

In Ethiopia, the demographic and health survey (DHS) program only includes subnational administrative levels like regions. In other words, the zones, Ethiopia's third administrative layer, were not the focus of the DHS program's design when providing accurate health-related information. For public health policymakers, research that only uses survey data at the lowest administrative levels, like zones, may not be reliable and practical since the estimates have large standard errors and coefficients of variation. As a result, additional sample data is needed to generate precise projections at the zone level [21]. We cannot increase the sample size due to financial and operational constraints.

There has been a growing demand for small area statistics in both the public and private sectors. Currently, SAE is experiencing high demand, with occasional nationwide spikes. In order to allocate resources and focus initiatives at lower-level administrations, SAE is a crucial technique for estimating health, education, and environmental indicators [23, 24]. The DHS program, the World Bank group, the International Monetary Fund (IMF), and the World Health Organization (WHO) were all very concerned about how to disaggregate DHS data and provide it to third-level government administrations [25–27]. The DHS host nations often request the DHS Program for subregional indicator estimates for development planning and policymaking [28]. In order to facilitate the mapping of health and economic inequality, the United Nations Population Fund (UNFPA) works to strengthen countries' capacities to produce and distribute high-quality, disaggregate data on population and development indicators, as well as demographic discrepancies, and to support humanitarian programming [29].

Estimating health-related parameters at unplanned geographic levels is crucial for successful health promotion and initiatives, improved healthcare delivery, and population-specific health planning and management. The lack of a readily available, adequate dataset for characteristics of interest in small areas has a detrimental effect on the ability of local and national organizations to manage significant health issues and associated risks in the community [12, 15, 18]. Developing a method for simulating reliable small-area statistics could solve this problem.

In the Ethiopian DHS, some zones have very few or no samples available. However, estimating the characteristics of interest, such as health insurance coverage, at the zone level for effective healthcare planning and resource allocation is crucial [30, 31]. To address this need, the study aims to estimate the level of health insurance coverage in Ethiopia at the zone level. The SAE approach's strength lies in using additional data sources, including census and administrative data [22, 32]. Therefore, this study used the HB approach to SAE, which combines data from the 2007 population census and the 2019 Ethiopian DHS.

Accurately estimating health insurance coverage at the zone level is vital in Ethiopia for several reasons. Firstly, it allows policymakers and healthcare providers to allocate resources more efficiently by providing precise information on the areas in need of healthcare services. Secondly, understanding the distribution of health insurance coverage ensures that healthcare services are accessible to all individuals, regardless of their location, thereby promoting equitable healthcare access. Thirdly, precise estimates of health insurance coverage at the zone level provide valuable insights for evaluating the effectiveness of existing policies and planning future interventions. Lastly, by estimating health insurance coverage at the zone level, healthcare providers can identify zones with high uninsured populations, which helps to design strategies to increase coverage, leading to a more efficient and equitable healthcare system. This knowledge can also help reveal geographic disparities and enable healthcare providers to address them more effectively [33, 34].

The Fay-Herriot model and its extensions have been used in various studies in the SAE framework. These include exploring spatiotemporal models using undernourished children in studies such as [34–36] and the Bayesian spatiotemporal approach to quantify SARS-CoV-2 [37]. Spatial SAE is also employed by [33, 38, 39]. In order to explore binary outcomes of response variables for SAE, a logit-normal model [40], logit-normal spatial model [41], HB logit-normal model [8, 42], HB logit-normal spatial model [43], zero-inflated Binomial [44], and zero-inflated negative Binomial model [45] are used under the Fay-Herriot model.

Although the SAE approach is popular in literature, it has yet to be widely practiced in Ethiopia. Therefore, this study aims to implement the HB SAE approach using the CBHI binary outcomes from the 2019 EMDHS and auxiliary variables from the 2007 population census datasets [46]. The main objective of this research is to estimate the community-based health insurance (CBHI) coverage at the zonal level in Ethiopia using the HB SAE approach. The study is expected to offer important insights into the spatial distribution of health insurance coverage, which is vital for making informed policy decisions and developing effective healthcare interventions at the zone level.

The paper is structured as follows: Section two explains the data sources, sampling design, and methods applied in the study, namely the Fay Herriot model under the different linking models and the HB SAE model. Section three presents the results and discussions, while Section four concludes.

## Methods and Materials

### Sampling design

The EMDHS sample for 2019 was stratified and chosen in two stages. At each lower administrative level, implicit stratification and proportional allocation were achieved by sorting the sampling frame inside each sampling stratum before sample selection, according to administrative units at various levels, and by employing a probability proportional to size selection at the first step of sampling. In the first step, 305 enumeration areas (EAs) were chosen proportional to EA size and with independent selection in each sampling stratum.

Each large EA chosen for the 2019 EMDHS was split to reduce the process of household listing. The survey was limited to one segment, with the likelihood proportional to segment size. Only households in the specified segment were listed; a 2019 EMDHS cluster is either an EA or a section of an EA. The second selection round involved selecting 30 households per cluster using an equal probability systematic selection from the freshly constructed household listing. All women aged 15–49 and men aged 15–59 who were either permanent inhabitants of the selected homes or guests who stayed in the household the night before the survey were eligible to be addressed [47].

### Data sources

This section describes the data sources used in this research. The 2019 EMDHS was the country's second mini-demographic and health survey. The first EMDHS is conducted in 2014. The response variable, community-based health insurance coverage, is taken from the 2019 EMDHS, whereas the auxiliary variables are from the 2007 population and housing census data. We assume that the functional relationship between health insurance and auxiliary variables remains constant across the survey and census periods.

The auxiliary variables for this study were derived from Ethiopia's population and housing census in 2007. The study considered 41 auxiliary variables. They were generated from the population census data in two ways: through variables relating to parents and young children under five. For example, children under five have been chosen based on their gender (male and female) and age (below one year, 1–2 years, and 4–5 years). On the other hand, the auxiliary variables of the parents included sex (male and female), place of residence (urban and rural), age (15–24, 25–34, 35–44, and 45–49), source of drinking water (improved and unimproved), educational levels (non-educated, elementary, secondary, and above), literacy (literate and illiterate), marital status (married, never married, and others), type of toilet facility (has toilet facility and does not have toilet facility), the number of sons who passed away (none, one, or two or more), the number of daughters who passed away (none, one, or two or more), the size of the family (less than five, and five or more), the presence or absence of disabilities (disabled and not), and the type of employment (government employment, private employment, self-employment, employer, unemployed, or other employment) [2, 48, 49]. These variables are considered at the zonal level as auxiliary variables in SAEs.

**Fig. 1:**
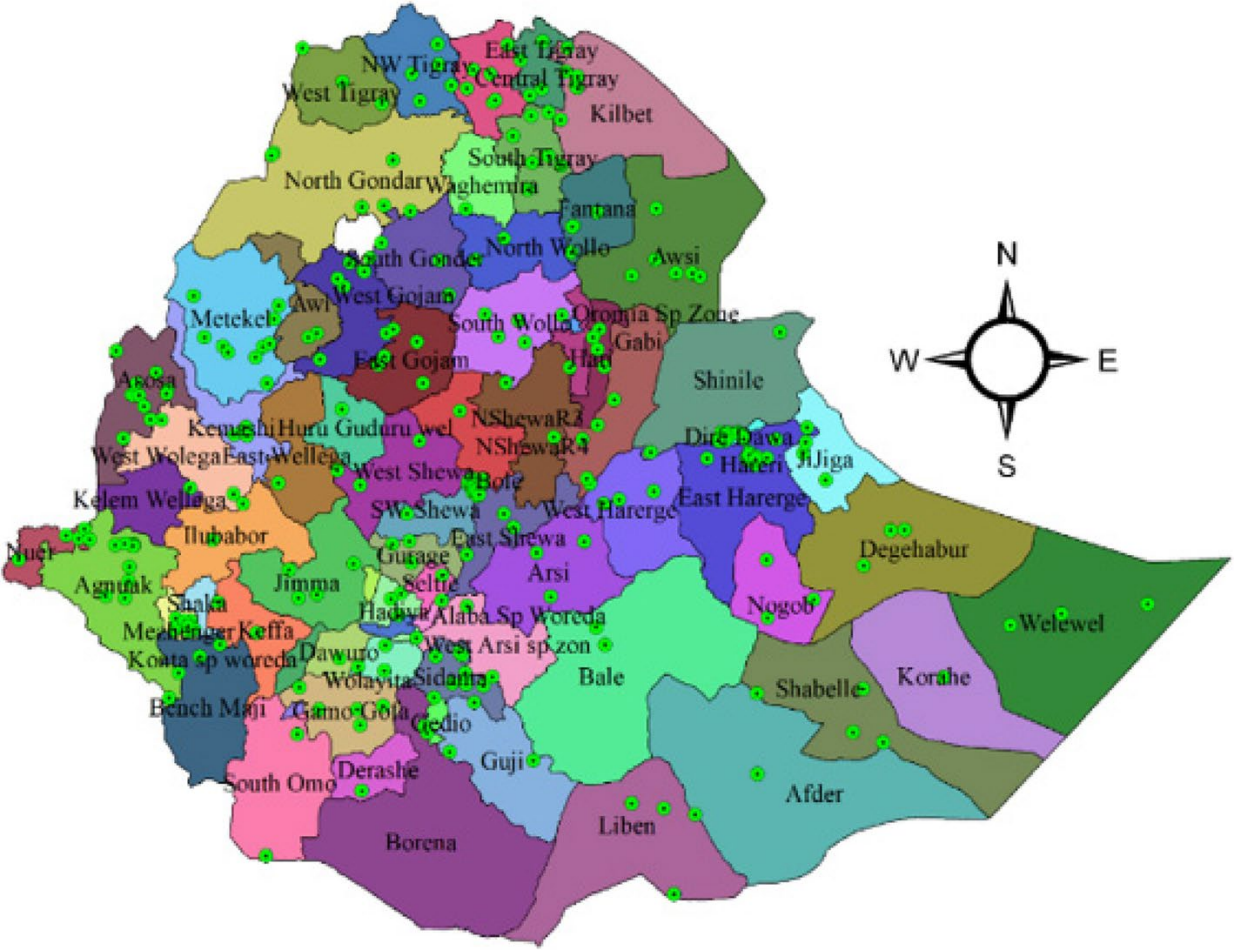
The enumeration area within Ethiopian zones

Figure 1 shows the Ethiopian zones with the 2019 EMDHS enumeration areas. Ethiopia has nine regions and two administrative cities at the subnational administrative level. However, there are 83 zones, of which 77 were sampled, and the remaining 6 were non-sampled zones for community-based health insurance coverage in the 2019 EMDHS data.

### The Fay-Herriot model

In 1979, Fay and Herriot introduced the Fay-Herriot model to estimate income in counties in the United States [23]. Since then, numerous researchers have explored the concepts of SAE in various ways. Several authors have written comprehensive textbooks on the SAE approach at both the unit and area levels [22, 50], with the most recent one discussing the concept of SAE in mixed-effect models [46]. Pfeffermann [32] conducted an excellent review of the new developments in SAE.

The most popular small-area model for area-level data is the Fay-Herriot model [23, 24], which includes an area-linking model. In order to compare binary data, we looked at three popular models. The Fay-Herriot model [23] is the first with known sampling variances and normal distributions for both the sampling and linking models. The second model is the log-normal model, and the third is the normal-logistic model. The normal-logistic model differs from the Fay-Herriot model only in the linking model by using a logit-normal distribution instead of a normal distribution. The log and logit link functions transform the health insurance coverage data in the linking model.. The logit linking model is used here to ensure that the estimates fall within the proper range of (0, 1).

There are two models in a general area level model. A sampling model for the sampling error of direct survey estimates and the linking model linked the population value for area-to-area specific auxiliary variables $x_{i} = (x_{i1} ,x_{i2} , \cdots ,x_{ip} )$ through a logit model $\theta_{i} = x_{i} {{\varvec{\upbeta}}} + \nu_{i}$, where $\nu_{i}$ the random area effects are the true means of the target variable in the m areas $i = 1,2, \cdots ,m$.

Consider simple random sampling within areas ($i = 1, \cdots ,m$) and sample proportion $\overline{y}_{i}$ as direct estimators (i.e., estimators from the 2019 EMDHS data only) of the population mean. Let $N_{i}$ be the population sizes of an area $i$ of finite population N. And let $y_{ik}$ be the binary response characteristics of interest (i.e., households with and without health insurance coverage) for a unit $k$ in the area $i$. The small area proportion parameter that needs to be estimated is given by $P_{i} = \sum {y_{ik} } /N_{i}$, and the variance $P_{i}$ is denoted by $\Psi_{i}$ and given by $\Psi_{i} = Var(P_{i} ) = \frac{{P_{i} (1 - P_{i} )}}{{N_{i} }}$.

The response variables $y_{i}$ for the $i^{th}$ area response variable are CBHI enrolment. The variables are binary, which are CBHI users and non-users. Thus, the appropriate binomial distribution is $y_{i} \sim bin(N_{i} ,P_{i} )$, where $P_{i}$ are the observed local zones CBHI prevalence in the zones ($i = 1,2, \cdots ,m$), $y_{i}$ the observed number of CBHI enrolments in the zone $i$, and finally $N_{i}$, the sample sizes at the zone $i$. The logistic regression model, which is the binomial regression model, is appropriate to predict $\theta_{i}$. Thus, the predictive *logit* regression model is given by $\theta_{i} = logit(P_{i} ) = {\mathbf{x\beta }} + {{\varvec{\upnu}}}_{{\mathbf{i}}}$ [8, 41, 44]. The three commonly used models are summarized as.

- **Model 1** (Normal Fay-Herriot model)
- Sampling model: $$y_{i} /\theta_{i} \sim^{iid} N(\theta_{i} ,\Psi_{i} )$$.
- Linking model: $$\theta_{i} /{{\varvec{\upbeta}}},\sigma_{\nu }^{2} \sim^{iid} N(x_{i} \beta ,\sigma_{\nu }^{2} )$$.
- **Model 2** (Normal-log Fay-Herriot model)
- Sampling model: $$y_{i} /\theta_{i} \sim^{iid} N(\theta_{i} ,\Psi_{i} )$$.
- Linking model: $$log(\theta_{i} )/{{\varvec{\upbeta}}},\sigma_{\nu }^{2} \sim^{iid} N(x_{i} \beta ,\sigma_{\nu }^{2} )$$.
- **Model 3** (Normal-logistic Fay-Herriot model):
- Sampling model: $$y_{i} /\theta_{i} \sim^{iid} N(\theta_{i} ,\Psi_{i} )$$.
- Linking model: $$logit(\theta_{i} )/{{\varvec{\upbeta}}},\sigma_{\nu }^{2} \sim^{iid} N(x_{i} \beta ,\sigma_{\nu }^{2} )$$.

The sampling variance $\Psi_{i}$ is the direct estimator estimated from the survey data. Model 1 is a matched model since the sampling and linking models can be combined to produce a simple linear mixed model. However, a nonlinear linkage model also unmatched model, such as model 2 or model 3, is generally recommended for modeling proportions [51, 52].

### Hierarchical Bayes SAE model

We employed the HB SAE approach by assigning prior distributions on unknown parameters $\beta$ and ${\sigma }_{\upsilon }^{2}$ to compute a posterior distribution of $\mu$, which produces the point estimator as well as credible intervals [46]. The parameter of interest $y_{i}$ and other model parameters are represented as random variables rather than fixed, unknown values under the Bayesian paradigm [51, 52]. Using Bayes' Theorem, this approach combines data-derived information from a likelihood function with previous knowledge about the parameter of interest and model parameters to produce a posterior distribution for the parameters.

Let $\mu = logit(\theta )$, and then the posterior distribution is

2.1 $$P\left(\mu , \beta ,{\sigma }_{\upsilon }^{2}|data\right)\propto P\left( data|\mu , \beta ,{\sigma }_{\upsilon }^{2}\right)P\left(\mu , \beta ,{\sigma }_{\upsilon }^{2}\right)$$

The HB approach also requires specifying prior distribution for population parameters ${{\varvec{\upbeta}}}$ model coefficients as

$$f({{\varvec{\upbeta}}}) \propto 1$$

For the between area variations parameters or model component parameters, the prior is considered the non-informative uniform distribution given as

$$f(\sigma_{\nu }^{2} ) \propto Unif(0,1)$$

We use Markov Chain Monte Carlo (MCMC) techniques to generate the model parameters. MCMC algorithms approximate posterior distributions by sampling from a probability distribution. In order to obtain the results of each HB model we fit, we run 50,000 sampling iterations (the length of each Markov chain) [53, 54]. One measure of model compatibility that can be used in evaluating the compatibility of Bayes statistics is the Deviance Information Criterion (DIC) [55]. The optimal candidate model is chosen using the DIC. It is interpreted similarly to the AIC, with models with smaller DIC being preferred. It is based on the model's deviation and penalized for model complexity.

The coefficient of variation (CV), which is displayed as a percentage of the estimate, shows the sampling variability. Large CVs are considered untrustworthy for small area estimates. If the CV for a small area estimate is unacceptably high, it will be deemed untrustworthy [56]. Although there are no globally recognized tables to determine what is "too large" [57], the smallest CV is preferred. The study uses the BayesSAE R software package [58].

Bias diagnostic measures are crucial in SAE to assess the accuracy of model-based estimates. One such measure is to plot the direct estimates on the y-axis and the model-based estimates on the x-axis. By examining the deviation of the small area estimates from the regression line fitted values, we can determine the level of bias.

### The Generalized Variance Function (GVF)

When dealing with a complex situation, the Generalized Variance Function (GVF) can be used to reduce the uncertainty in design-based variance estimates [20, 58, 59]. Generally, the standard design-based estimators tend to have high variability when the sample sizes are small. Therefore, having stable estimators is crucial for smaller domains or zones. The GVF provides more reliable estimators for domain-level variance than direct design-based variance estimators [60]. The GVF was fitted by using simple linear regression as

$${y}_{i}={\beta }_{0}+{\beta }_{1}{x}_{i}+{e}_{i}, i=1, 2, \cdots,m$$

Where the dependent variable $y_{i}$ is $log(Var(P_{i} ))$, and the independent variable $x_{i}$ is, the $log(P_{i} )$ success probability at the zone level. $\beta_{0}$ and $\beta_{1}$ are the constant and slope parameters estimates, and $e_{i} \sim^{iid} N(0,\sigma^{2} )$ therefore, the GVF function is $\widehat{GVF} = exp(\hat{\sigma }^{2} /2)exp(\hat{\beta }_{0} + \hat{\beta }_{1} x_{i} )$ [30, 59–61].

## Results

### Model selection and fitting

We used Ethiopia's population and housing census 2007 data to select suitable auxiliary variables for Fay-Herriot modeling [23, 62]. The auxiliary variables are used for linking to the response variable using the linking small area model. The response variable, community-based health insurance coverage in Ethiopia, is binary; therefore, data transformation is conducted. The log and logit transformations are used to transform the binary outcome variable.

There are almost 41 auxiliary variables available from the census data, and we have done exploratory analysis before selecting appropriate variables. Eight auxiliary variables were chosen to link the survey data using the appropriate variable selection method. We applied the DIC to select the most suitable candidate model for estimating health insurance coverage. The smaller the deviance is, the better the model. The logit transformations of the Fay-Herriot model have small DIC. Thus, the logit Fay-Herriot model is the best for analyzing health insurance coverage Table 1.

**Table 1:** Model comparison

Model	DIC
Fay-Herriot	1133.3222
Log Fay-Herriot	1133.3750
Logit Fay-Herriot	1130.0822

### The generalized variance function

**Fig. 2:**
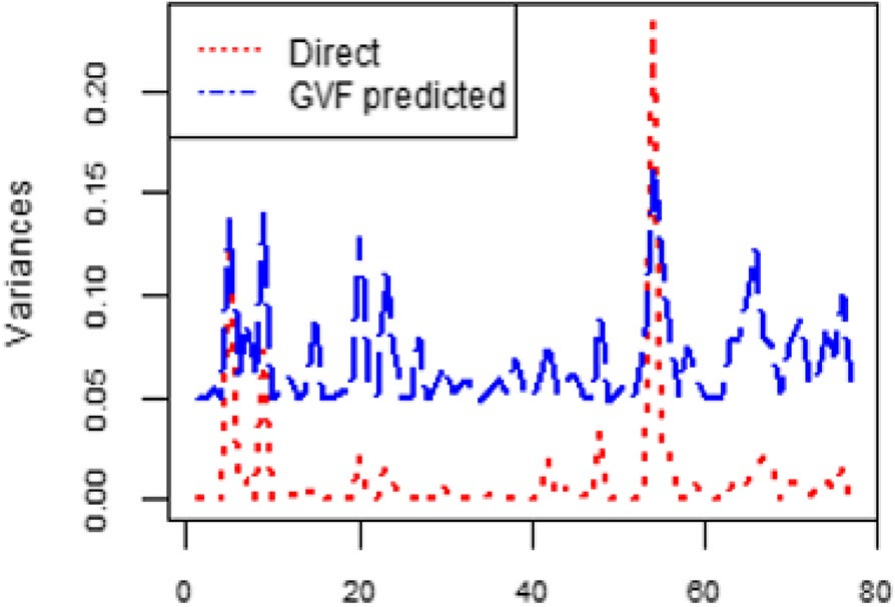
Dispersion plots of GVF fit for health insurance coverage

Standard design-based estimators are not precise enough when sample sizes are too small. In this study, the GVF was used to produce area-level variance estimators that would be more stable [63]. The GVF aids in smoothing out unreliable and noisy estimated variances [59, 60]. Figure 2 shows the direct survey estimated variance (red color) and the GVF predicted variance (blue color). The GVF predicted variance is relatively smoother than the direct survey variances.

The table (Table 2) displays the posterior mean, standard deviation (SD), and 95% credible interval (CI) for the regression coefficients of the normal-logistic Fay-Herriot model. It is evident from the table that marital status (married) and having more than five children are significant predictors of health insurance coverage since the CIs of both variables do not include zero.

**Table 2:** The 95% CIs for the regression coefficients under the normal-logistic Fay-Herriot model

				95% credible interval	
Coefficient	Auxiliary variables	Posterior mean	SD	Lower limit	Upper limit
$\beta_{0}$	Constant	-2.75	22.53	-46.43	41.00
$\beta_{1}$	Age category more than 44 years	-24.43	13.41	-50.51	2.73
$\beta_{2}$	Urban	-0.40	1.49	-3.29	2.52
$\beta_{3}$	Female	-5.13	3.50	-11.99	1.80
$\beta_{4}$	Not disabled	14.29	22.93	-31.21	58.92
$\beta_{5}$	Married	-4.56	2.28	-9.07	-0.095
$\beta_{6}$	Nunber of children more than five	4.19	1.73	0.75	7.65
$\beta_{7}$	Government employement	-5.40	3.59	-12.44	1.64
$\beta_{8}$	Primary level of education	-0.68	2.09	-3.39	4.75

### Bias diagnostic

**Fig. 3:**
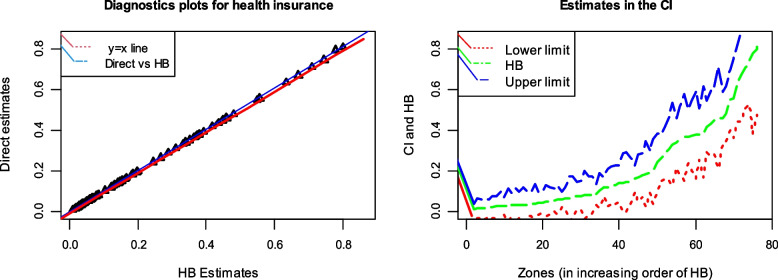
Bias diagnostics plots (left) and zonal-wise 95% CIs for HB estimates (right)

The bias diagnostic measure assesses the validity, while the 95% CI and coefficient of variation (CV) assess the precision of the model-based estimates [57]. The regression of the direct estimates on the true population values is projected to be linear with the identity line since they are unbiased estimators of the population parameters. Suppose the model-based small area health insurance estimates are close to the population's true values. In that case, the regression of direct estimates on model-based small area estimates should be comparable. As a result, we plotted the direct and model-based estimates on the y and x axes, respectively, and looked at how much the small area estimates deviated from the regression line fitted values. Figure 3 shows the estimates of HB bias diagnostics plots against the baseline $y = x$.

**Fig. 4:**
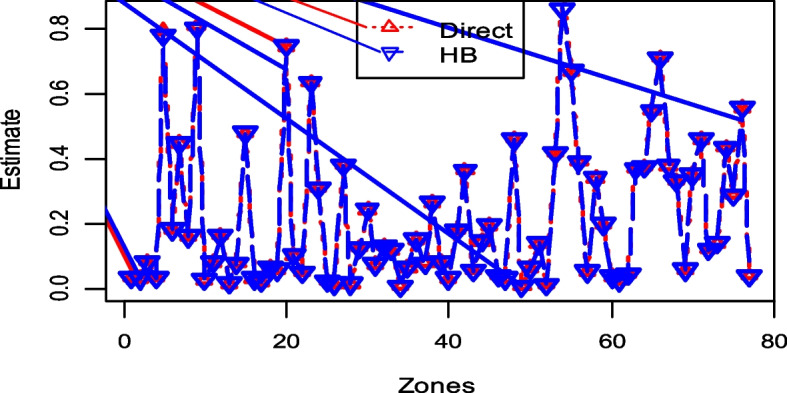
The consistency of HB and the direct survey estimates

Figure 4 displays the direct survey and model-based HB estimates of the prevalence of CBHI in Ethiopian zones. In principle, the values of HB and direct survey estimates are equivalent [21]. Therefore, HB and direct survey estimates are comparable and consistent for all zones (Fig. 3).

**Fig. 5:**
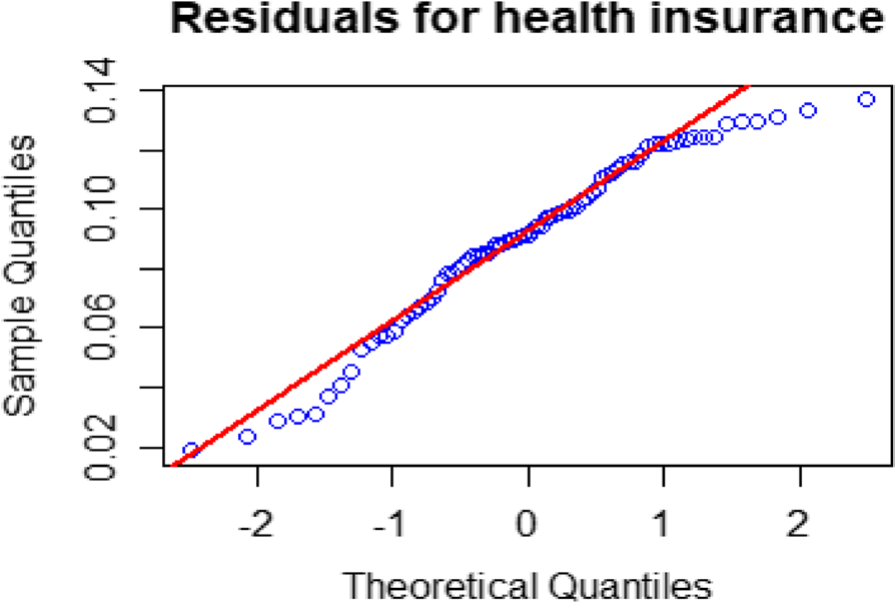
Q-Q plot of the Fay-Herriot model residuals

The Bayesian model assumes that the random area-specific effects are normally distributed with a zero mean and constant variance $\sigma_{\nu }^{2}$. If the underlying model assumptions hold, the zonal level residuals should be distributed randomly around zero. To test the normality assumption of the residuals of the fitted model, we used the normal probability (Q-Q) plot. Figure 5 displays the Q-Q plots of the zonal-level residuals for health insurance coverage. The Shapiro–Wilk test was used to assess the normality assumption of the zonal-specific random effects, and it was found to meet the criteria for the assumptions.

### Improvements of model based HB estimates of health insurance coverage

**Fig. 6:**
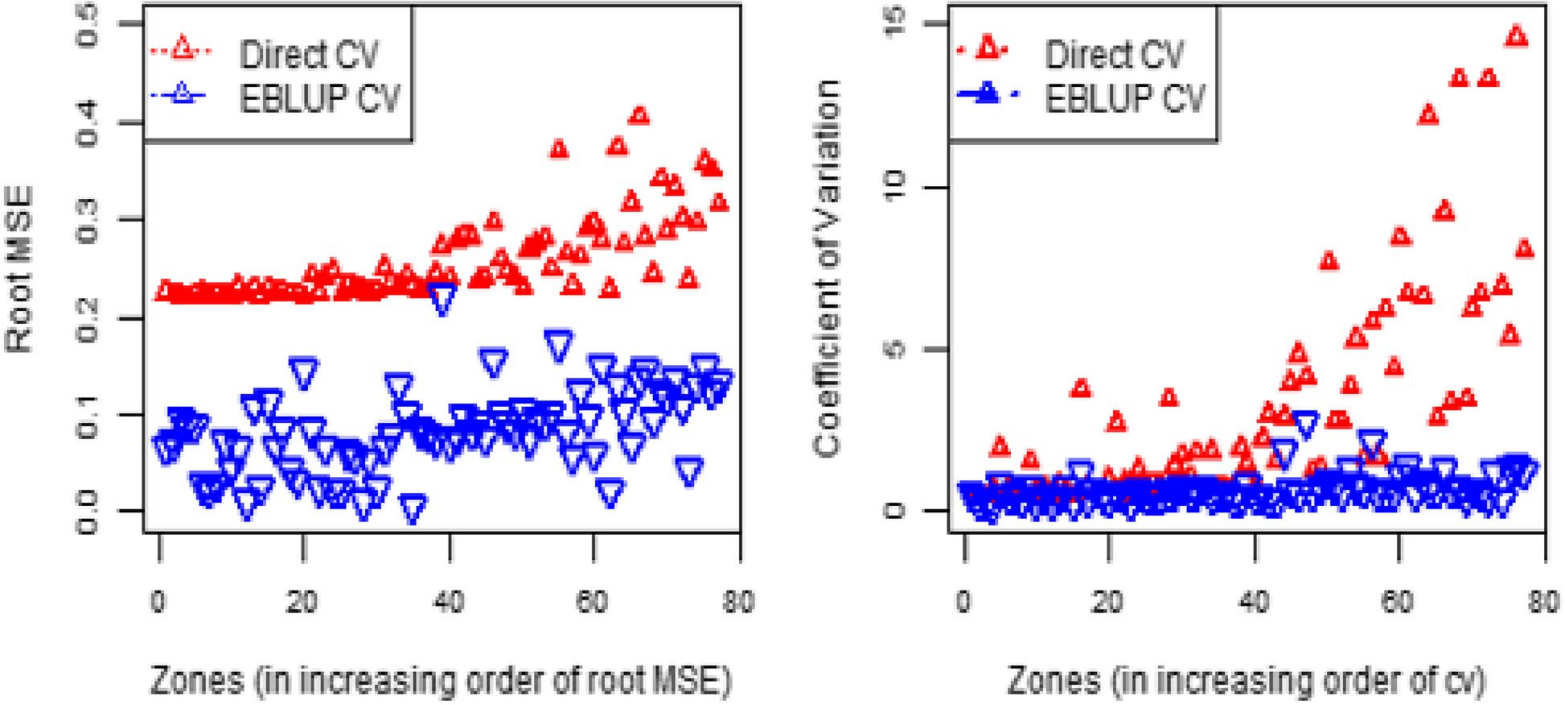
Zone-specific root MSE (left) and percentage CV (right) of direct and HB health insurance coverage estimates

The root MSE and CV of the HB estimates for the prevalence of health insurance coverage are shown in Fig. 6. The CVs and root mean square error (root MSE) of the HB estimates were smaller than the corresponding direct survey estimates. These findings demonstrate that HB estimates, which borrow strength from auxiliary variables in the census data, are more accurate than direct survey estimates [32, 64]. Model based HB estimates are empirical best linear unbiased predictors (EBLUP).

Table 3 shows the efficiency improvements in the CV of HB estimates over the direct survey estimates. The minimum, first quartile, mean, third quartile, and maximum values for the CV for HB estimates are found to be lower than those for the equivalent direct survey estimates. As a result, compared to estimates from the direct survey, those from the HB Fay-Herriot model are more accurate and reliable. Compared to direct survey estimates, CV efficiency has increased by a mean of 46.75 and a maximum of 70.95. The HB estimates had an advantage over direct survey estimates because they borrowed strength from the census data source.

**Table 3:** Comparatives of % CV for the direct and HB estimates of health insurance coverage

Quantities	Direct CV	EBLUP CV	Efficiency gain
Minimum	0.2337	0.2338	-6.03
1st quartile	1.8876	1.4892	21.11
Median	5.9076	3.3748	42.87
mean	6.8314	3.6376	46.75
3rd quartile	8.9087	4.3265	51.43
Maximum	15.6256	4.5400	70.95

Due to the zero sample size, no direct survey estimates of health insurance coverage for non-sampled zones exist. However, based on the HB approach and auxiliary data from census data, we provided an estimate for health insurance coverage. Table 4 displays the CV of HB estimates for small area health insurance for non-sampled zones.

**Table 4:** The CV of health insurance coverage for non-sampled zones

Value	Direct CV	EBLUP CV
Minimum	-	0.5600
1st quartile	-	2.1563
Median	-	3.2135
Mean	-	3.7568
3rd quartile	-	4.9320
Maximum	-	5.1223

**Fig. 7:**
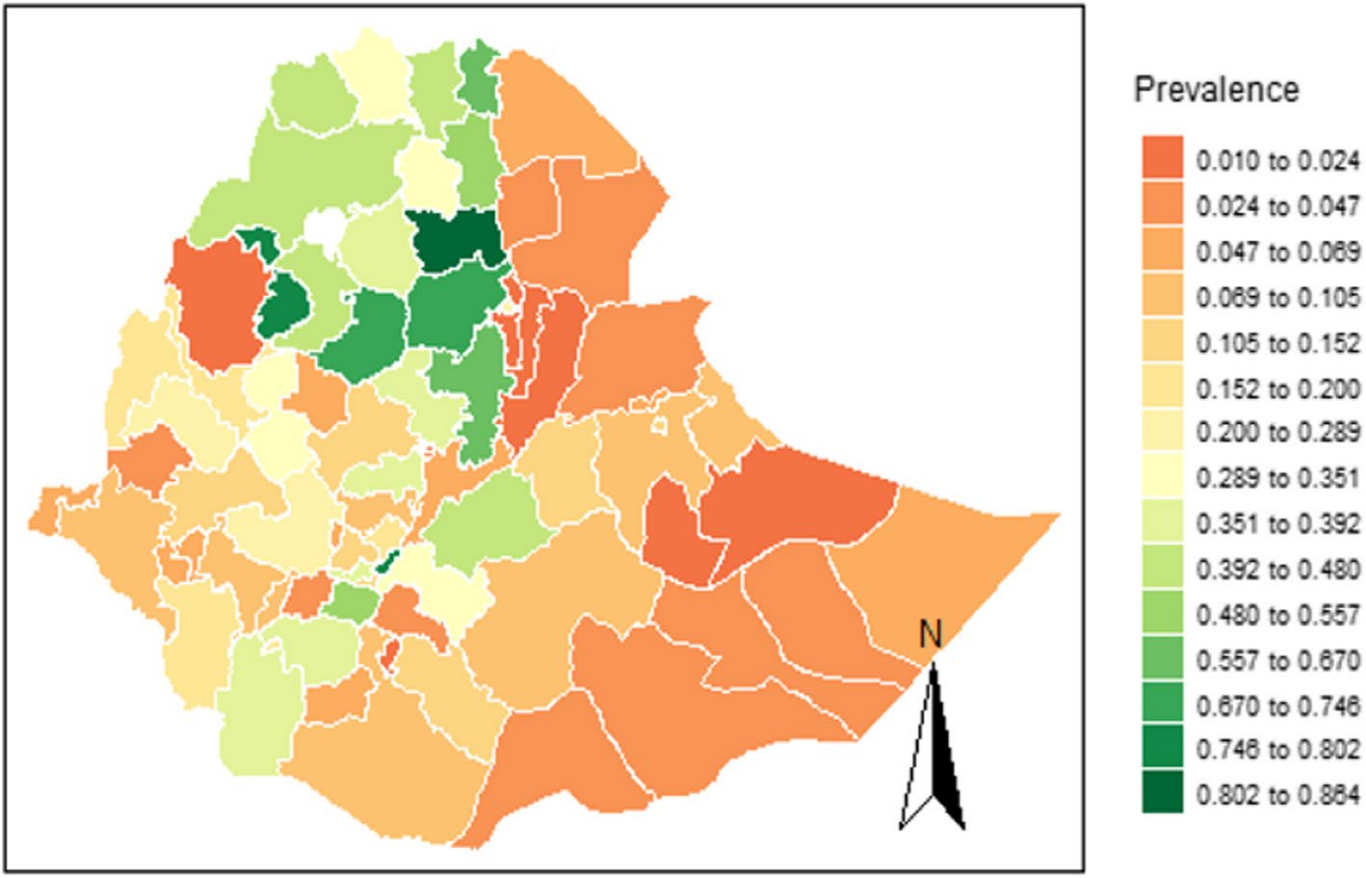
The distribution of the HB estimates for the prevalence of health insurance coverage across Ethiopian zones

Figure 7 depicts the prevalence of health insurance coverage across Ethiopian administrative zones. On the Ethiopian maps, the prevalence indicates that green indicates better and red indicates inadequate health insurance coverage. Zones such as North Wollo, Awi, East Gojam, South Wollo, and East Tigray, located in the country's north and northwest, have better estimates of the prevalence of health insurance coverage. However, coverage for health insurance needs to be improved in the Metekel, all Afar and Somalia regional zones, Kelem Wollega, Gedio, Shaka, Mezhenger, Sidama, Horo Guduru Wollega, and Dawro zones.

## Discussion

This research focused on estimating the zone-level CBHI in Ethiopia by utilizing the 2019 EMDHS and the 2007 population census data. The CBHI enrollment status is represented using binary outcomes, where zero indicates non-insured individuals and one represents those enrolled in CBHI [65]. For binary outcomes, we employed the Logit Normal model with the HB SAE method [8, 22, 41, 50]. This method is advantageous because it simplifies model specifications and is relatively easy to compute using the MCMC technique [44]. This study utilizes the GVF to effectively smooth unreliable and noisy estimates of sampling error variances for SAE obtained through direct surveys. The results indicate that GVF helps to smooth out unreliable and noisy estimated variances, which is consistent with previous SAE literature [59, 60, 65–67]. Figure 2 shows the direct survey estimated variance (red color) and the GVF predicted variance (blue color). The GVF predicted variance is smoother compared to the variances obtained through direct survey estimates.In HB SAE applications, model diagnostics for small area estimates are typically employed. These techniques are used to verify the assumptions made by the model [59, 68]. The Shapiro–Wilk normality test and Q-Q plot are the most commonly used methods to establish normality [53, 69]. The model assumptions of the random area effect $\nu$ are assumed to have identically and independently distributed with mean zero and variance ($\sigma_{\nu }^{2}$ = 0.3243). The results of the Shapiro–Wilk test indicate that the residuals at the zonal level (W = 0.950 and *p*-value = 0.085) support the normality assumption for the data on health insurance coverage. This suggests that the normality assumption for the health insurance coverage data is adequately met. Similar studies in Ethiopia about children under the age of five have agreed with the normality assumptions [31, 34].

The bias diagnostics plot is the second diagnostic measure used to evaluate the reliability of HB estimates [68]. It helps identify potential sources of bias and assesses the model's validity. When estimating health insurance coverage through HB, it should be comparable to direct survey estimates. Figure 3 shows the direct survey estimates on the y-axis and the HB estimates on the x-axis. The graph depicts a strong relationship between both estimates, which is consistent with various literature sources [33, 53]. The direct survey-based and HB estimates agree, indicating that they are consistent and aligned. To put it differently, the estimates obtained from the HB method and the direct survey method are consistent with each other.

Additionally, three widely used diagnostic measurements, such as percent CV, root MSE, and 95% credible intervals, are also used to assess the accuracy of the HB estimates [69]. Even though there are no precise cutoff values for CV to determine precision, the lower the CV, the more precise and consistent the estimates [70]. According to the condensed tables and figures (Fig. 6 and Table 3), the HB estimates have a lower CV in each zone. The HB estimates are relatively more accurate than the corresponding direct estimates in all zones, according to the CV, root MSE, and 95% CI, which are consistent with the different study results [57, 71, 72].

The results of this study found that auxiliary variables such as marriage and the number of children of more than five are statistically significant predictors of CBHI prevalence. This finding explicitly revealed that respondents had more than five children in the household, which is a significant effect of the CBHI scheme. Other studies are consistent with our results [3, 73, 74]. The number of children over five significantly impacts the CBHI prevalence, which is consistent with the studies [2, 3]. Similarly, the studies conducted in South Gondar zones of Ethiopia show that those having more than five children in the household were two times more likely to use the CBHI scheme when compared with those who have fewer than five children [2, 3].

This could be because families with more children require more medical care, such as immunizations, which could explain why they seek enrollment in CBHI programs. However, recent research contradicts earlier studies in Kenya, which suggested that the number of children did impact CBHI enrollment [75]. Under UHC, healthcare should be accessible to all without causing financial hardship [17]. Achieving UHC is essential for long-term development and reducing poverty. It is also critical in any effort to combat social inequality and improve access to healthcare [5, 16].

The study also demonstrates that auxiliary variables, like marital status, can significantly influence the possibility of CBHI adoption in Ethiopia. This suggests that evaluating and enhancing the uptake of CBHI schemes requires an understanding of the demographic and social factors of the population. This finding aligns with a study in the North Shoa Zone of Ethiopia by [76], which also found a significant association between marital status and CBHI utilization.

Using the estimation from the disaggregated level HB estimates, we were able to identify disparities in CBHI coverage between Ethiopian administrative zones. It was found that the Amhara and Tigray regional zones have better CBHI coverage than other regions of the nation, as shown in Fig. 7. This is because the Ethiopian government approved and initiated the CBHI practice in the Tigray and Amhara regions before rolling it out nationwide to improve healthcare quality [49]. As a result, the prevalence of CBHI in Ethiopia differs from region to region, with higher enrollment rates in the Amhara and Tigray regions than in others [77]. This finding agrees with a study by [2], which also reported higher enrollment rates in these regions. Similarly, another study by [48] found that the overall pooled prevalence of CBHI enrollment in Ethiopia was 45.5%, with better coverage in the Amhara and Tigray regions. Our research also confirms that there are variations in health insurance coverage across different administrative zones [77, 78]. It is evident that health insurance coverage varies geographically.

Our study is unique in that it provides synthetic estimates for small areas with zero sample sizes. These estimates are generated by either combining data from different sources or using statistical models to infer values for smaller areas based on larger, more reliable datasets. Synthetic estimates help make informed decisions when direct measurements are unavailable [22, 50]. In Table 4, the HB model-based SAE produced CBHI estimates for non-sampled zones. We used the HB model-based SAE to estimate CBHI coverage in non-sampled zones, which borrowed auxiliary variables from census datasets. This approach is consistent with most studies in SAE contexts [39, 78, 79].

## Conclusions and policy implications

The SAE approach is a statistical method used to estimate the proportion of socioeconomic indicators at the local or domain level. This method has been extensively studied and adopted as a target by national statistics offices in developed countries. However, there is a need for more research on how to apply SAE in developing countries such as Ethiopia. For example, in order to track progress towards achieving the SDGs, it is essential to have disaggregated estimates at lower administrative levels or specific domain levels. This initiative aligns with the global framework of indicators for monitoring the SDGs, which suggests that information should be disaggregated not only geographically (in subregions such as provinces, municipalities, or districts) but also by income group, gender, age, race, ethnicity, immigration status, and disability status [80].

This study found that by combining data from the 2019 EMDHS with additional information from the 2007 population census, more accurate estimates of health insurance coverage at the zone level were obtained using the SAE approach. The research also suggests that relying solely on survey data for estimating the CBHI coverage in Ethiopia's administrative zones is less precise than when auxiliary variables are also taken into account.

Ethiopia is strongly committed to achieving UHC, which means providing high-quality healthcare services that are accessible and equitable to all. Our study has highlighted the differences across Ethiopian zones regarding their coverage of CBHI schemes. We have also identified some areas with high coverage and some with low coverage. This information can be very useful in improving local planning and policy decision-making, which can ultimately lead to an increase in the coverage of CBHI schemes across Ethiopia.

Overall, this study stands out due to its innovative approach of integrating data from two sources, namely the 2007 population census and the 2019 EMDHS, to improve the accuracy of health insurance coverage estimation at the zone level. The study employed the HB modeling framework in SAE to estimate health insurance coverage at the lower administrative level (zones), which can help policymakers gain a more nuanced understanding of coverage disparities and target resources and interventions more effectively. Furthermore, this paper's novelty lies in estimating the non-sampled zones, which will receive equal attention from policymakers as the sampled zones. This study contributes to evidence-based decision-making in healthcare planning and resource allocation, making it valuable for researchers and policymakers.

### Summary of key policy implications

The study's key policy implications are summarized below:

(i) Policymakers can gain valuable insights into the spatial distribution of health insurance coverage at the zone level. The map in Fig. 7 can identify zones with inadequate coverage, which can help policymakers prioritize interventions, allocate additional funding, and implement specific outreach programs to increase health insurance enrollment in those zones.
(ii) The study highlights disparities in health insurance coverage across Ethiopia. Policymakers can use this information to improve coverage in underserved areas with targeted interventions such as financial incentives, awareness campaigns, and improved access. Ensuring equity in access and benefits for all populations is key, which aligns with the UHC.
(iii) The study offers a robust methodology to estimate health insurance coverage at lower administrative levels, which are zones in the Ethiopian case. The methodology can be replicated to monitor coverage changes and evaluate policy interventions. Policymakers can use the findings to set targets, monitor progress, and make informed decisions about health insurance expansion.
(iv) The study emphasizes the significance of having trustworthy and current data sources for precisely estimating health insurance coverage. Policymakers can use this research to advocate for better data collection efforts, including regular surveys and updates to census data. By investing in data infrastructure and ensuring data quality, policymakers can monitor and evaluate programs more effectively, identify areas for improvement, and make data-driven policy decisions.

## Data Availability

The 2019 DHS data for this study was received through a registration request from the DHS program website, https://www.dhsprogram.com. The GPS enumeration area shapefiles were received similarly from https://www.dhsprogram.com. Shapefiles for Ethiopian zonal administrative borders are also accessible on the website https://africaopendata.org. For research purposes, the Ethiopian Central Statistical Agency gives access to a 10% sample of the 2007 census.

## Abbreviations

CBHI: Community-based health insurance
CI: Credible interval
CV: Coefficient of variation
DHS: Demographic and health survey
EA: Enumeration areas
EBLUP: Empirically best lieaner unbiased predictors
EMDHS: Ethiopian mini DHS
GVF: Generalized variance function
HB: Hierarchical Bayes
MCMC: Morkov Chain Monte Carlo
MSE: Mean square error
SAE: Small area estimation
SDG: Sustainable development goal for health
SD: Standard deviation
UHC: Universal health coverage
